# Partially Neutralizing Potency against Emerging Genotype I Virus among Children Received Formalin-Inactivated Japanese Encephalitis Virus Vaccine

**DOI:** 10.1371/journal.pntd.0001834

**Published:** 2012-09-27

**Authors:** Yi-Chin Fan, Jo-Mei Chen, Hsien-Chung Chiu, Yi-Ying Chen, Jen-Wei Lin, Chen-Chang Shih, Chih-Ming Chen, Chao-Chin Chang, Gwong-Jen J. Chang, Shyan-Song Chiou

**Affiliations:** 1 Graduate Institute of Microbiology and Public Health, College of Veterinary Medicine, National Chung Hsing University, Taichung, Taiwan; 2 Department of Periodontology, School of Dentistry, National Defense Medical Center and Tri-Service General Hospital, Taipei, Taiwan; 3 Department of Neurology, Mennonite Christian Hospital, Hualien, Taiwan; 4 Division of Infectious Disease, Department of Internal Medicine, Tungs' Taichung MetroHarbor Hospital, Taichung, Taiwan; 5 Arboviral Diseases Branch, Center for Disease Control and Prevention, Fort Collins, Colorado, United States of America; Centre for Cellular and Molecular Biology, India

## Abstract

**Background:**

Genotype I (GI) Japanese encephalitis virus (JEV) that replaced GIII virus has become the dominant circulating virus in Asia. Currently, all registered live and inactivated JEV vaccines are derived from genotype III viruses. In Taiwan, the compulsory JEV vaccination policy recommends that children receives four doses of formalin-inactivated Nakayama (GIII) JEV vaccine.

**Methodology/Principal Findings:**

To evaluate the influence of genotype replacement on the post-vaccination viral neutralizing ability by GIII and GI viruses, the small panel of vaccinated-children serum specimens was assembled, and the reciprocal 50% plaque-reduction neutralizing antibody titers (PRNT_50_) were measured against Nakayama vaccine strain, CJN GIII human brain isolate and TC2009-1 GI mosquito isolate. The seropositivity rate (PRNT_50_≥1∶10) and geometric mean titers (GMT) against the TC2009-1 virus were the lowest among the three viruses. The protective threshold against the CJN and TC2009-1 viruses could only be achieved when the GMT against Nakayama virus was ≥1∶20 or ≥1∶80, respectively. Using undiluted vaccinees' sera, the enhancement of JEV infection in K562 cells was observed in some low or non-neutralizing serum specimens.

**Conclusions/Significance:**

Our preliminary study has shown that neutralizing antibodies, elicited by the mouse brain-derived and formalin-inactivated JEV Nakayama vaccine among a limited number of vaccinees, have reduced neutralizing capacity against circulating GI virus, but more detailed studies are needed to address the potential impact on the future vaccine policy.

## Author Summary

Genotype I (GI) Japanese encephalitis virus (JEV) that replaced GIII virus has become the dominant circulating virus in Asia; however, all available JEV vaccines are derived from genotype III viruses, and no study has been conducted on the cross-neutralization and protection elicited by GIII JEV vaccines against GI viruses using vaccinated children’s serum specimens collected from the general population. Genotype I virus was first detected in Taiwan in 2008, and became the dominant circulating JEV, and was island-wide within a year. In the present study, the small panel of GIII virus vaccinated-children serum specimens were not only showed lower strain-specific neutralization against GI virus as compared to the GIII vaccine and human isolates but also observed the enhancement of GI virus infection in K562 cells in some low or non-neutralizing serum specimens. These preliminary results indicated the reduced neutralization potency due to genotype replacement should be closely monitored in the JE epidemic/endemic regions in the future.

## Introduction

South and Southeast Asia are Japanese encephalitis (JE) endemic areas in which approximately 10% of the susceptible populations are infected with JE virus (JEV) each year, based on the ratio of asymptomatic to symptomatic infections of 200 to 1 [1], [2], [3]. The most cost-effective control strategy for JE is vaccination, and there are several licensed vaccines, including live-attenuated, chimeric live-attenuated and inactivated SA14-14-2; inactivated Nakayama; P3 and Beijing-1 vaccines [3], [4], [5], [6]. In Taiwan, compulsory vaccination has been implemented since 1968 using the mouse-brain derived and formalin-inactivated Nakayama vaccine, and since then clinical JE cases have decreased dramatically to 20–30 cases each year [7]. It has been estimated that vaccine effectiveness is in the range of 85% to 90% after immunization with two doses of inactivated Nakayama vaccine [7], [8].

We have witnessed dramatic changes in the molecular epidemiology of circulating JEV in the past two decades. Historically, genotype III (GIII) viruses were the most widely distributed JEV in South and Southeast Asia [9]. However, genotype I (GI) JEV, having emerged in the 1970s in Thailand/Cambodia, has replaced GIII as the dominant circulating virus in JE endemic/epidemic regions since the 1990s [10]. Genotype I viruses first appeared in Japan, and by the 1990s the majority of Japanese JEV isolates belonged to GI [11]. Subsequently, the phenomena of genotype replacement were observed in many countries, including Korea, Vietnam, Thailand, and China [12], [13], [14]. Genotype I JEV was first detected in Taiwan in 2008, and became the dominant circulating genotype island-wide within a year [15], [16].

The nucleotide and amino acid variation between the envelope (E) glycoproteins of GIII and GI JEV is in approximately 12% and 3%, respectively [9]. All licensed JEV vaccine strains, including SA14-14-2, Nakayama, P3, and Beijing-1, belong to GIII. The reduced capacity of neutralizing antibody against field-isolated GIII viruses had been reported among vaccinated human serum samples [17], [18]. Thus, strain-specific neutralizing antibodies elicited by GIII JEV vaccines in vaccine recipients need to be assessed against GI virus. The protective efficacy of inactivated JE-VAX (suckling mouse brain-derived Nakayama vaccine) and P3, and live-attenuated SA14-14-2 vaccines has been evaluated in a mouse model. Beasley *et al.* have shown that mice that received JE-VAX vaccine or were passively transferred JE-VAX-vaccinated mouse sera had lower neutralizing antibody titers and were less protected against GI virus as compared to GIII virus, but the strain-dependent protection could not be excluded [19]. However, Liu *et al.* showed that the live-attenuated SA14-14-2 and inactivated P3 vaccines protected vaccinated mice equally against GIII and GI viruses [20]. In a series of *ex vivo* studies, Van Gessel *et al.* eloquently demonstrated that mice receiving passively transferred immune sera collected from adult human volunteers vaccinated with JE-VAX or SA14-14-2-derived IC51 (tissue culture-derived inactivated) vaccine were protected against GIII and GI viruses, and firmly established for the first time that the strain-specific correlate of protection (PRNT_50_≥10) could be accurately estimated by measuring the reciprocal neutralizing antibody titer at the time of viral challenge [21].

The cross-neutralization and protection elicited by GIII JEV vaccines against GI viruses are not consistent in mouse models. More importantly, no study has been conducted using vaccinated children's serum specimens collected from the general population. In the present study, the panel of specimens collected from children immunized with mouse brain-derived and formalin-inactivated Nakayama vaccine in Taiwan were assembled and used to assess the potency of neutralizing antibodies against the vaccine strain, a GIII local human isolate, and a newly introduced GI mosquito isolate.

## Materials and Methods

### Amino Acid Analysis

The nucleotide and amino acid sequences of JEV GI, GIII, and Nakayama viruses were retrieved from GeneBank and analyzed using BioEdit version 7.1.3 [16], [22]. To localize the amino acid substitutions, the protein structure of JEV E glycoprotein was downloaded according to recent report [23] and analyzed by Swiss-Pdb Viewer 3.7 structure analysis software. The antibody-accessible amino acids should have at least a 35% surface accessibility potential based on the result of structure analysis [24]. In order to assess the impact of amino acid substitutions on the impact of E protein, the stability calculation was performed for all amino acid substitutions by using the Prediction of Proteins Mutations Stability Changes server (http://babylone.ulb.ac.be/popmusic/index.html).

### Study Subject

In Taiwan, a compulsory vaccination program was implemented in 1968 which utilizes mouse-brain derived and formalin-inactivated Nakayama vaccine. Children receive an initial dose of vaccine at one-and-one-half-years of age, and subsequent doses two weeks later, one year later, and in the first grade of elementary school. We also collected serum samples from children who received varying numbers of doses of vaccine and at different time post final booster vaccination.

### Ethics Statement and Human Sera

All the serum samples used in this study were collected from an already-existing collection in two hospitals, the Mennonite Christian Hospital in Hulian and the Tungs' Taichung Metroharbor Hospital in Taichung, in 2010 and were anonymized. For serum sample collection, the clinical protocols were reviewed and approved by the institutional review boards of these two hospitals (10-03-007-ER and 99006). Serum was obtained from whole blood after clotting at room temperature and then centrifuged at 3,000 rpm for 10 min. The aliquot of serum was stored at −20°C until use.

### Serological Assay

Five JEV strains were used in the serological assay, including: the GIII, cluster III Nakayama vaccine strain; the GIII, cluster I CJN strain isolated from human brain in 1998; the GIII, cluster II T1P1 strain isolated from a mosquito pool in 1997 [15]; and the GI, cluster I TC2009-1 strain and GI, cluster II YL2009-4 strain isolated from mosquito pools in 2009 [16]. Viruses were amplified in C6/36 mosquito cells and stored in aliquots at −80°C until use.

Plaque reduction neutralization tests (PRNT) are the most suitable method for assessing neutralizing antibodies against JEV. The PRNT protocol used is similar to that in our previous report with some modification [17]. The BHK-21 cells were dispensed into each well of 6-well polystyrene plates (Costar, Cambridge, MA, USA). The plates were incubated at 37°C for 36 h to form a monolayer. Serum samples were inactivated at 56°C for 30 min before a two-fold serial dilution was carried out. A target dose of about 100 plaque forming units (PFUs) of JEV were then incubated with the previously diluted serum samples. The mixture of diluted test serum and control virus was added onto the BHK-21 monolayers. After adsorption for 1 h at 37°C, the infected cells were overlaid with 4 ml/well of 1.1% methyl cellulose (Sigma) in MEM containing 2% FBS and 1% antibiotics. After an additional incubation for 3.5–4 days, the cells were fixed with 10% formalin and stained with 1% crystal violet. The PRNT titer was obtained from the reciprocal of the dilution of serum that reduced the plaque number by at least 50% relative to the virus-only control.

To determine the potential for antibody-dependent enhancement (ADE) of different genotypes of JEV, K562 cells were used to measure viral yield and infection rate [25]. The undiluted serum samples were used in the assay. Briefly, 100 µl serum was mixed with 2×10^5^ K562 cells and 2×10^4^ PFUs virus (MOI = 0.1), and incubated with gentle rotation at 37°C for 2 h. After being washed twice, the cells were incubated with RPMI-FBS medium at 37°C for one day. The culture supernatants were collected and the viral yield was determined by plaque count using BHK-21 cell monolayers; also, infected K562 cells were collected, stained with mouse anti-JEV HIAF, and the cell infection rate was estimated by flow cytometry. The monoclonal antibody 4G2 (obtained from Dr. Chang GJ of US CDC, Fort Collins, CO) was diluted 1∶100 and was used as the positive ADE control.

### Statistics

A cut-off value to determine seropositivity of neutralizing antibody titer was defined as PRNT_50_≥10. Seropositive subjects were defined as those having a reciprocal antibody titer above or equal to the cut-off value; seronegative subjects, those falling below the cut-off value. Antibody titers below the cut-off value of 1∶10 were given an arbitrary value of 5 for geometric mean titer (GMT) calculation.

The cut-off value for virus yield and cell infection rate for differentiating neutralization and enhancement was calculated from the average of five repeat measurements plus two times the standard deviations (SD) of the viral yield and cell infection rate obtained with the negative controls. In the negative controls, RPMI-FBS was substituted for the serum specimen in the virus–serum–cell preparation. Virus yield and cell infection rate of at least 2SDs above the negative control was determined to represent a statistically significant event.

Student's t rest was used for all analyses, and statistical significance was defined as a P value <.05.

## Results

### Amino Acid Analysis

**Figure 1:**
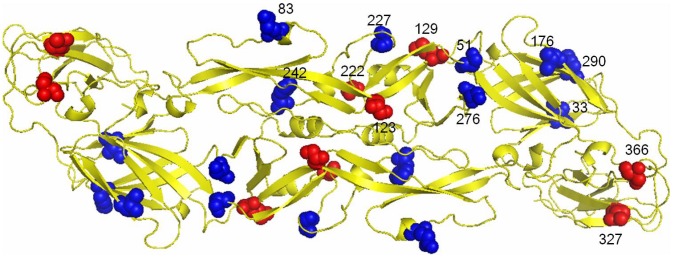
Structural locations of Japanese encephalitis virus (JEV) envelope (E) protein variations. Amino acid variations between the Nakayama vaccine strain and the GIII brain-isolated CJN strain are indicated in blue, and variations between the Nakayama vaccine strain and the GI field-isolated TC2009-1 strain are indicated in both blue and red.

The GIII Nakayama strain has been used exclusively for the manufacture of formalin-inactivated suckling mouse brain-derived human vaccine in Taiwan since 1968. An extensive field investigation and molecular epidemiological study outlined in our previous report indicates that the replacement of JEV GIII by GI occurred in 2009 in Taiwan [16]. JEV envelope (E) protein is the primary antigen eliciting protectively neutralizing antibodies. The amino acid differences in the E protein region between Nakayama vaccine strain and GI and GIII sequences of virus isolated from Taiwan are shown as Table S1. Amino acid differences in the E protein between the vaccine strain and strains used in this study are highlighted in Figure 1. There are eight amino acid differences between Nakayama and the human brain isolate GIII CJN virus, including E protein amino acid positions 33, 51, 83, 176, 227, 242, 276, and 290 (Figure 1). Among these residues, position 33, 176, and 290 are located in E domain I (EDI); 51, 83, 227, 242, and 276 are located in EDII; and none are in EDIII. There are 13 amino acid variations between Nakayama and mosquito isolate GI TC2009-1 virus, including E protein amino acid positions 33, 51, 83, 123, 129, 176, 222, 227, 242, 276, 290, 327, and 366 (Figure 1). Among these residues, positions 33, 176, and 290 are located in EDI; 51, 83, 123, 129, 222, 227, 242, and 276 are located in EDII; and 327 and 366 are located in EDIII. The unique differences distinguish the CJN and GI strains from the vaccine Nakayama strain were at E protein amino acid positions 33, 51, 83, 176, 227, 242, 276, and 290.

### Strain-Specific Neutralizing Antibody

A pilot experiment was conducted to determine the difference of neutralizing antibody titers against five JEVs, GIII Nakayama (cluster III), GIII T1P1 (cluster II), GIII CJN (cluster I), GI TC2009-1 (cluster I), and GI YL2009-4 (cluster II), were evaluated using the small panel of serum samples (Figure S1) [15], [16]. The correlation of PRNT_50_ between T1P1 and CJN or TC2009-1 and YL2009-4 was 0.75 and 0.92 (Figure S1), respectively. Due to insufficient amount of some serum samples, the GIII Nakayama, GIII CJN, and GI TC2009-1 viruses were selected as viral strains for further analysis.

**Figure 2:**
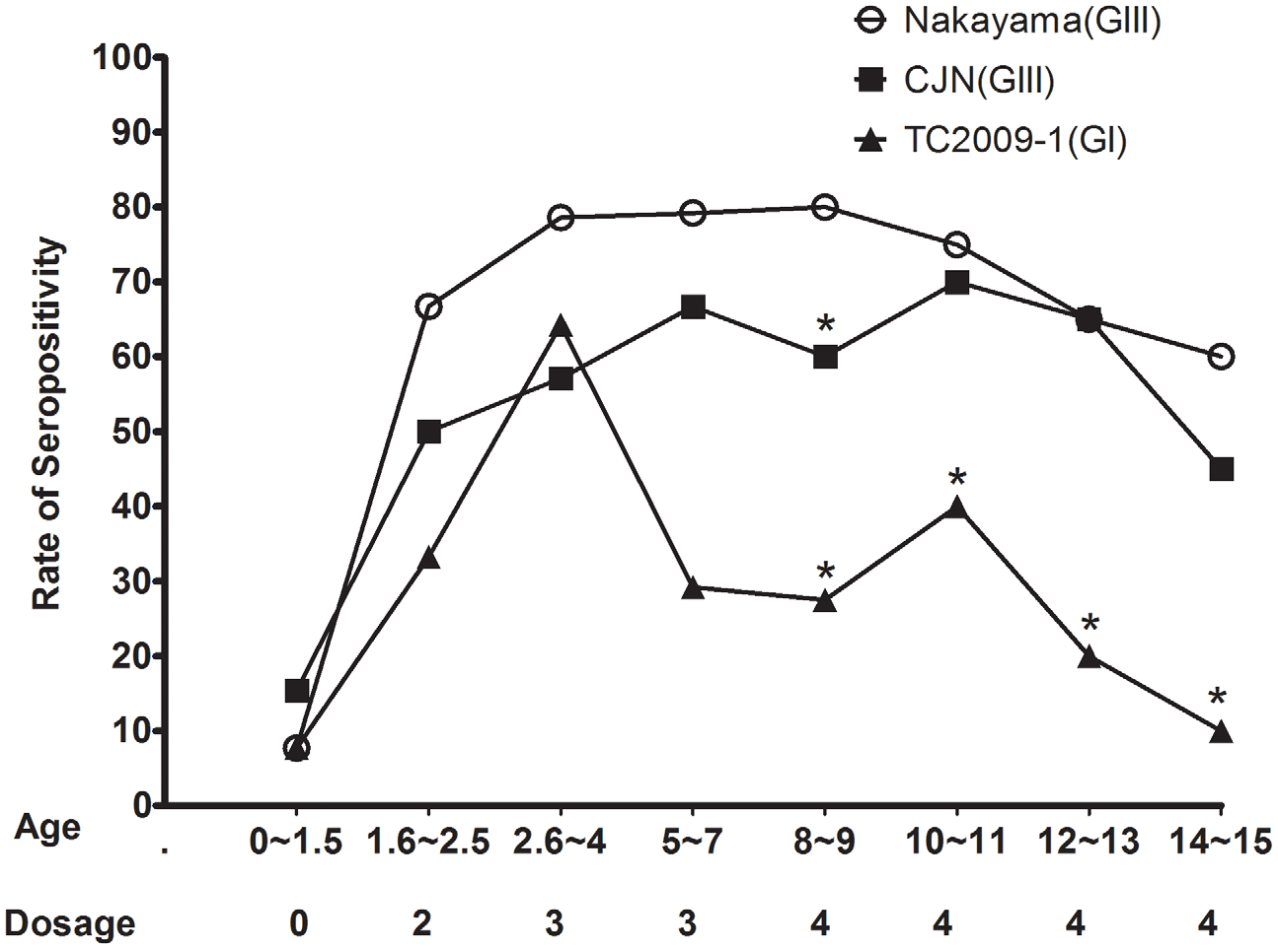
The seropositivity rate of children immunized with inactivated Nakayama JEV vaccine against JEVs. Seropositivity is defined by a reciprocal titer of ≥10 in a 50% plaque-reduction neutralization test using BHK-21 cells. Significantly different (P<.05) seropositivity rates against CJN or TC2009-1 as compared to that for Nakayama virus are indicated by asterisks.

A total of 157 serum samples from vaccinated children were collected and grouped based on age (0–15 years) and dosage of vaccination (0–4 doses) at the time of sampling (Table 1). The seroprotection or seropositivity rate, defined by the PRNT_50_≥10 using BHK-21 cells against homologous Nakayama strain, was 66.7% after primary vaccination, peaking after the receipt of the 4^th^ dose of vaccine (80.0% for 8–9-years-old), but decreased gradually to 60.0% in children aged 14–15 years (Figure 2). The trend in strain-specific seroprotection rate against the GIII CJN strain was similar to that of Nakayama virus, but the seroprotection rate themselves were generally lower (ranged from 0 to 21.5%) as compared to those for the Nakayama strain. The seroprotection rate against GI TC2009-1 virus were much lower as compared to other two viruses, especially in the 14–15-years-of-age group (P<.05, Figure 2).

**Table 1:** Geometric mean titers (GMT) of strain-specific neutralizing antibody against vaccine and field-isolated genotype III and I viruses among serum samples collected from children immunized with inactivated JEV Nakayama vaccine.

Age (years)	Sample size	Vaccination (Dosage)	PRNT_50_ GMT against virus (95% CI)		
			Nakayama	CJN	TC2009-1
0–1.5	13	0	5.9(4.2–8.3)	5.9(4.6–7.5)	5.6(4.4–7.0)
1.5–2.5	6	2	25.2(4.9–129.4)	8.9(4.4–18.2)	9.9(2.3–42.8)
2.5–4	14	3	38.0(15.5–93.0)	18.1(7.5–43.9)	15.6(7.1–34.5)
5–7	24	3	15.9(10.8–23.2)	12.6(8.8–18.1)	**6.5(5.4–7.8)**
8–9	40	4	13.9(10.8–17.9)	**9.7(7.8–11.9)** 1	**6.6(5.7–7.7)**
10–11	20	4	16.8(10.1–28.1)	20.0(11.0–36.2)	**8.1(5.7–11.5)**
12–13	20	4	13.2(8.6–20.2)	12.3(8.3–18.3)	**5.9(5.0–7.1)**
14–15	20	4	9.3(6.9–12.5)	7.3(5.9–9.1)	**5.4(4.8–5.9)**

1 Boldface indicates titers significantly different (P<.05) from those for Nakayama virus by pairwise comparisons.

The geometric mean titers (GMT) of strain-specific PRNT_50_ against the GIII vaccine and CJN viruses and the TC2009-1 GI virus are shown in Table 1. The GMT of neutralizing antibodies against homologous Nakayama strain was 25.2 after primary vaccination and reached a peak (38.0) following the third dose of vaccine, but decreased gradually after the final booster and dipped below the protective threshold of 10 in children aged 14–15 years. The strain-specific GMT of neutralizing antibodies against the GIII CJN strain trended similarly to those against the Nakayama virus, but the titers were lower. Interestingly, the GMT of antibodies neutralizing field-isolated GI TC2009-1 virus were significantly lower than those for the other two viruses (P<.05) and the titers were below the presumptive protective threshold of 10 with the exception of children aged 2.5–4 years who had received a third dose of vaccine.

### Protective Threshold for GI Virus

Neutralizing antibodies elicited by the mouse brain-derived, formalin-inactivated Nakayama vaccine could be protective against circulating GIII and GI virus in Taiwan (Figure 2 and Table 1). Serum specimens capable of neutralizing Nakayama virus were selected and stratified into groups with PRNT_50_ titers of 10, 20, 40, 80, 160, and ≧320. The strain-specific GMT from each group was calculated for the human brain GIII CJN and mosquito GI TC2009-1 viruses (Table 2). The grouping results suggest that the GMT reach the presumptive protective threshold (PRNT_50_ = 10) against CJN and TC2009-1 viruses when the neutralizing titer against Nakayama virus is 1∶20 or 1∶80, respectively.

**Table 2:** Strain-specific protection threshold of inactivated Nakayama Japanese encephalitis virus vaccine.

PRNT_50_ against Nakayama	Sample size	PRNT_50_ GMT against virus (95% CI)	
		CJN	TC2009-1
10	37	9.09 (7.8–10.64)	5.28 (4.97–5.65)
20	34	15.01 (12.02–18.75)	6.63 (5.74–7.7)
40	18	20.76 (13.46–31.92)	9.25 (7.34–11.69)
80	6	31.71 (6.61–151.36)	11.21 (5.50–22.91)
160	3	63.41 (1.77–2243.88)	31.71 (4.36–229.61)
≧320	4	67.22 (4.24–1066.60)	113.00 (59.43–211.84)

### Potential of Antibody-Dependent Enhancement of Infection

**Figure 3:**
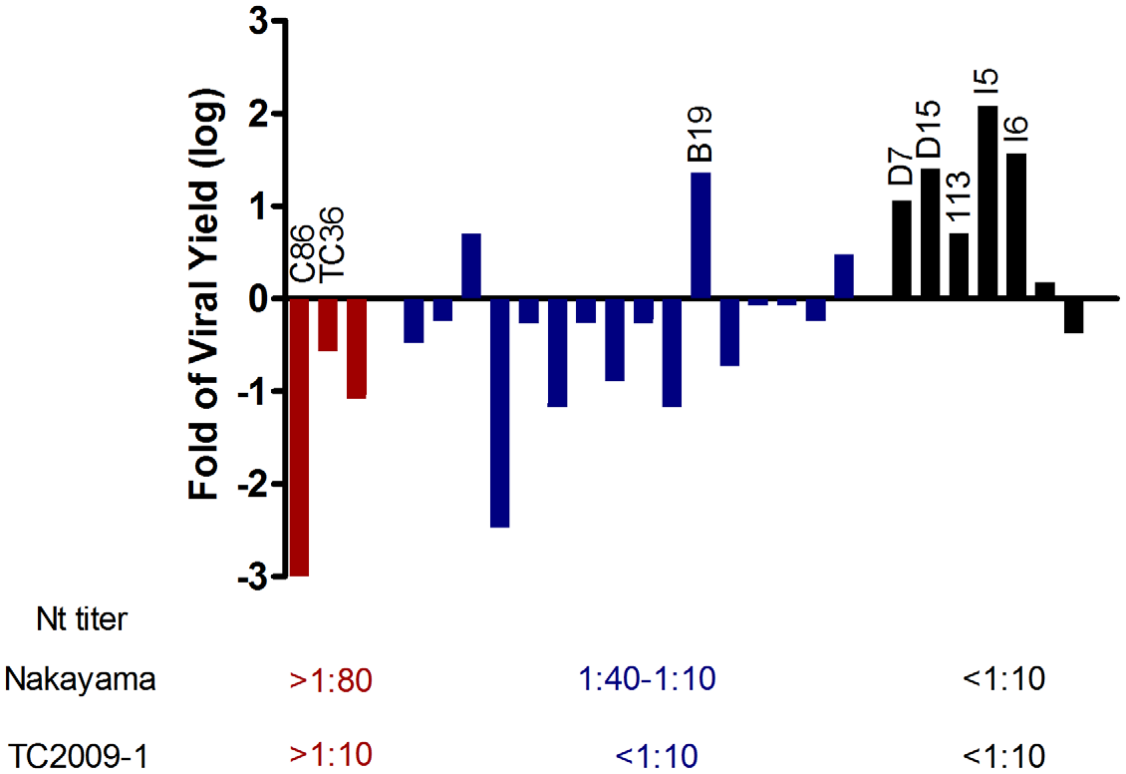
Effects of children's undiluted serum samples on viral yield of GI JEV from K562 cells. The magnitude of enhancement of viral yield is calculated as follows: Log_10_ (serum-treated viral titer/untreated viral titer). The K562 cells infected with labeled samples C86, TC36, B19, D7, D15, 113, I5 and I6, were subjected to infection rate analysis by flow cytometry (Figure 4).

Antibodies elicited by the inactivated Nakayama vaccine are less potent in neutralizing GI virus, and the majority of vaccinees' PRNT_50_ titers are below the protective threshold (Table 2). The enhancement of virus infection resulting from vaccination has been suggested [26]. Thus, 26 serum samples from vaccinated children were selected, and the undiluted serum samples were used to estimate the potential for ADE of GI JEV infection in K562 cells by measurement of viral yield (Figure 3). The three serum samples most highly neutralizing against Nakayama (PRNT_50_≧80) also strongly inhibited the infectivity of GI JEV. Of the 16 weakly neutralizing serum samples (PRNT_50_ = 10 to 40 against Nakayama), only three exhibited some increase in virus yields as compared to the control serum. However, most (6/7) of the non-neutralizing serum samples (PRNT_50_<10 against Nakayama) enhanced GI virus infection.

**Figure 4:**
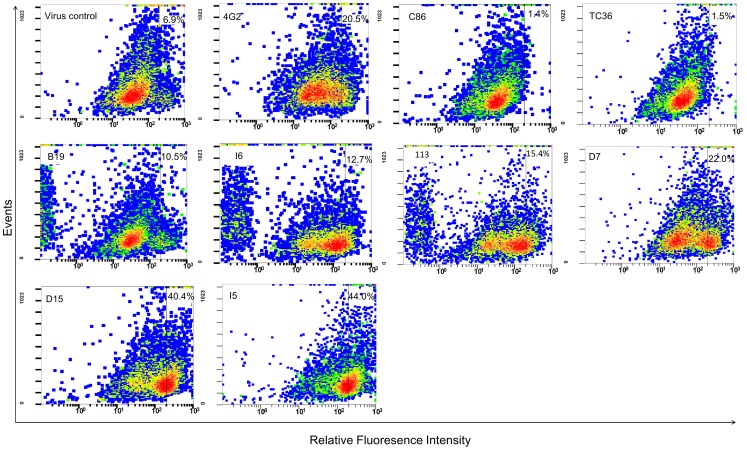
Patterns of ADE in K562 cells infected by virus treated with serum samples. The virus control is GI TC2009-1 virus-infected K562 cells in the absence of serum samples. The ADE of TC2009-1 virus in K562 cells by using 1∶100 diluted 4G2 monoclonal antibody as a positive ADE control and the serum samples collected from vaccinated children including C86, TC36, B19, I6, 113, D7, D15, and I5. The K562 cells were collected, stained with mouse anti-JEV HIAF, and analyzed for infection rate by flow cytometry.

The risk of ADE of GI JEV infection in K562 cells was also analyzed by flow cytometry to determine the infection rate of cells infected with serum-treated virus. The results of selected samples, not all samples were included due to insufficient amount individual serum, are shown in Figure 4. At an MOI = 0.1, the untreated K562 infection rate by TC2009-1 was 6.9%. Thus, an infection rate of less than 6.9% can be interpreted as neutralization, and greater than 6.9% as enhancement. The flavivirus group-cross reactive murine monoclonal antibody 4G2, used as an ADE control at a 1∶100 dilution, resulted in an infection rate of 20.5%, significantly higher than that of the virus control. The T36 (PRNT_50_ = 20) and C86 (PRNT_50_ = 160) samples neutralized GI virus, and the cell infection rate were reduced to 1.4–1.5%, significantly lower than that of the virus control (P<0.05). The B19 serum neutralized Nakayama and CJN viruses, but not TC2009-1 virus. Treatment with B19 serum resulted in some degree of enhancement of TC2009-1 infection (10.5% infection) (P>.05). Sera I6, 113, D7, D15, and I5, non-neutralizing against all three viruses, showed significant enhancement of GI JEV infection (P<.05).

In addition, we conducted an *ex vivo* ADE experiment with vaccinees serum samples, and the result was shown in Supplemental Figure 2. Due to the availability of vaccinee's serum, the undiluted serum from TC36, C86, D7, and I5 only were preincubated with 1000 PFU of TC2009-1 virus and injected intraperitoneally into suckling mouse (four mice per serum sample). The brain of infected suckling mouse was collected at four days post-inoculation; and the virus titer was determined by the plaque forming assay in BHK21 cells. The TC36 and C86 serum samples almost completely neutralized TC2009-1 virus replication in suckling mouse. However, compared to virus control, the mouse brain virus titers were significantly increased to 5.53- and 5.79-fold when the TC2009-1 virus was pre-incubated with D7 or I5 serum samples, respectively. The result of this *ex vivo* ADE experiment was consisted with the result of flow analysis (Figure 4).

## Discussion

Long-term evolution, geographical barriers and host-parasite interactions have resulted in many flaviviruses evolving into multiple genotypes, including dengue virus serotypes 1 through 4, West Nile virus, and JEV [27], [28], [29], [30], [31], [32]. Genotype replacement in dengue virus serotypes 3 and 4 in Sri Lanka and Puerto Rico, respectively, has resulted in increased transmissibility and epidemic potential of the new viruses [33], [34]. Vaccination is the most effective strategy to control flavivirus epidemics [35]. Genotype I JEV has replaced GIII as the dominant genotype throughout Asian countries, and human clinical cases due to GI virus infection have been observed in China [10], [36]. The GIII mouse brain-derived, inactivated Nakayama vaccine and live-attenuated SA14-14-2 vaccine are the most widely distributed vaccines in Asian countries, including Japan, Korea, Taiwan, Vietnam, Thailand, Malaysia, China, India, and Nepal [3], [4]. The replacement of GIII by GI virus provides the opportunity to evaluate the contribution of genotype replacement to the strain-specific vaccine effectiveness of JEV. The reported protective efficacies of GIII JEV vaccines against GI virus are not consistent due to differences in evaluation models used, including virus strain, passive or active immunization, and challenge dosage (17, 18, 19).

The Nakayama JEV was isolated in 1935, and the vaccine derived from it was developed in 1956 [37]. Differences between vaccine strain and circulating GIII viruses are expected, but the vaccine is proven to be highly protective [38]. The efficacy of formalin-inactivated Nakayama JEV vaccine in vaccinees who received one, two, and three doses of immunization is 85.59%, 91.07% and 98.51%, respectively [8]. The trial of mouse brain-derived inactivated JEV vaccine, including Nakayama or Nakayama plus Beijing-1 strains, was conducted in Thailand in 1984–1985, and the estimated efficacy was 91%. But, several JE-confirmed cases were diagnosed among vaccinated group indicating the potential of primary vaccine failure. The JEV genotype I was first isolated in the Southeast Asian countries; it has been suggested that the switch from genotype III to I in the 1980s might contribute to the primary vaccine failure without confirmed virological evidence [13], [39]. Our study also showed that the vaccine, used in Taiwan, offered strain-specific neutralization against field-isolated GIII CJN virus. This result suggests and supports that the amino acid variations, located in EDI and EDII, between these two viruses, are not critical in eliciting neutralizing antibody as compared to residues located in domain III [40].

Our previous report demonstrated GI JEV replaced GIII in Taiwan in 2009, and here we conducted the first study using serum samples from vaccinated children to systematically evaluate strain-specific neutralizing antibodies elicited by the GIII Nakayama vaccine [16]. Previous report indicated that the GMT titers against GIII isolates, including T1P1, CC27, CJN, and CH1392, were 2-fold lower than that against Nakayama strain; and Shyu et al., reported that only 37.9% of vaccinees sera in the 15–19 year-old group can actually neutralize JE5 Taiwanese isolate [17], [18]. In presented study, the neutralizing titer against GI virus was 8-fold lower (Table 2) than against Nakayama strain, and the seroprotection threshold against GI virus was 10% of vaccinees in the vaccinated 14–15 year-old group. Thus, the lower titers of antibody against GI in general in Taiwan might relate directly to overall antigenic variability between GIII and GI, but strain-dependent neutralization could not be totally excluded. The seroprotection rate should be appropriately estimated against currently circulating JE strains rather than against vaccine strain.

There were only five informative amino acids variations between GIII and GI JEV in the E protein: residues 123, 129, 222, 327, and 366. However, the antibodies elicited by the GIII JEV vaccines were only weakly neutralizing against circulating GI virus as compared to the human GIII isolate. Three of the five amino acid differences occur in domain II, which is involved in weakly or non-neutralizing antibody binding [41], and residues 129 and 222 are not accessible for antibody binding. Thus, the remaining two amino acid variations, residues 327 and 366, might play an important role in lower strain-specific neutralization against GI virus as compared to the GIII vaccine and human isolates. These two residues are accessible for antibody binding and are located in domain III of the E protein, which has been shown to be the most important region eliciting neutralizing antibody. Antibodies targeting domain III of E protein make up a relatively small proportion of the polyclonal human antibody response against flaviviruses, thus more detail studies are need to clarity the role of residues 327 and 366 [42].

GI virus-specific residues at position 327 and 366 located in the BC and DE loops on domain III of E protein, respectively, may play an important role in eliciting genotype-specific neutralizing antibodies. Genotype- and strain-specific neutralizing MAbs have been characterized against dengue virus serotypes 1, 2 and 3 [36]–[38]. Monoclonal antibody E104, derived from mice immunized with GII of DENV-1 virus, is a genotype-specific MAb, which recognizes residues 328, 330, 361 and 362 in the BC and DE loops of EDIII. Residues 328 and 329, located in the BC loop, are recognized by another DENV-3-derived genotype-specific MAb. Thus, the amino acids located in the BC and DE loops on domain III of E protein may involve in the induction of genotype-specific neutralizing antibodies.

Non-neutralizing antibodies are a possible risk factor for ADE in dengue pathogenesis during infection with heterologous or homologous dengue viruses, but the role of ADE contributes to JE disease is unclear [43]. Vaccine-induced enhancements of virus infection have been documented for members of different virus families as well [26]. Our preliminary *in vitro* and *ex vivo* studies suggest that the potential of ADE with vaccinees serum specimens may be increased due to GIII to GI replacement. Prior to the genotype replacement in Taiwan, the average mortality rate of confirmed cases of JEV infection was 7.8% (2000–2008). However, during 2009–2010, after GI JEV became the dominant circulating virus, the average mortality rate increased to 14.2% (Official statistics of the Department of Health, Taiwan). The neurovirulence of GI and GIII viruses was similar and might not directly associate with an increasing case fatality rate of JE cases [19], [20]. The potential for ADE, measured by *in vitro* assays and supplemented by the limited number of *ex vivo* assays, increases dramatically when the neutralization titer of vaccinee serum decreases to below the protective threshold of 1∶10 against the vaccine strain. The statistical differences we have seen in older children, reflecting waning of neutralizing antibody to GI virus to less than seroprotective threshold (Figure 2). The presence of memory B cells, CD4+ and CD8+ T cells, and anamnestic response has been indicated after received JEV inactivated vaccine [44], but rapidly decline of seroprotection rate against field-isolated JEVs also has been suggested [45]. Thus, the duration of immunity among vaccinated adults should be evaluated comprehensively ten years after the final booster vaccination.

The conclusion of current study is limited by the small sample size and the volume of vaccinee's serum. With the Institution Approved Protocol in the further, we plan to increase the sample size and collect larger serum volume to increase more selection of GIII and GI strains in the analysis. The most cost-effective control strategy for JE is vaccination, but genotype replacement in JEV endemic/epidemic regions may reduce the efficacy of traditional GIII virus-based vaccines. The efficacy of GIII JEV vaccines should be closely monitored at the national or regional level. The potential impact due to genotype replacement could be overcome in the future by 1) increasing the effective immunogenic dose or incorporating a novel adjuvant in the vaccine formulation to improve the immunogenicity of the current vaccine, or 2) replacing the GIII vaccine strain with a dominant GI isolate and conducting a non-inferiority study of GI strain-specific immune responses.

## Supporting Information

Figure S1 **Selection of JEVs for neutralizing antibody assay.** Four JEVs, GIII T1P1 (cluster II), GIII CJN (cluster I), GI TC2009-1 (cluster I), and GI YL2009-4 (cluster II), were evaluated using a panel of serum samples (N = 10 and 15 for panel A and B, respectively) by a plaque-reduction neutralization assay. The correlations of PRNT_50_ were determined between (A) T1P1 and CJN or (B) TC2009-1 and YL2009-4. Number in black circle corresponding to the specimen number.
(TIF)

Figure S2 **Semi-ex vivo ADE experiment.** The undiluted serum was pre-incubated with 1000 PFU of TC2009-1 virus and inoculated intraperitoneally into four suckling mice per serum. Brains of inoculated sucking mice were harvested and virus titers determined by plaque forming assay in BHK-21 cells.
(TIF)

Table S1 **Amino acid variations among JEV E protein between genotype and cluster.**
(DOC)
