# Supplementary figures and images for: Partially Neutralizing Potency against Emerging Genotype I Virus among Children Received Formalin-Inactivated Japanese Encephalitis Virus Vaccine
Source: PLoS Negl Trop Dis. 2012 Sep 27;6(9):e1834. doi: 10.1371/journal.pntd.0001834 (PMC3459827; doi:10.1371/journal.pntd.0001834)

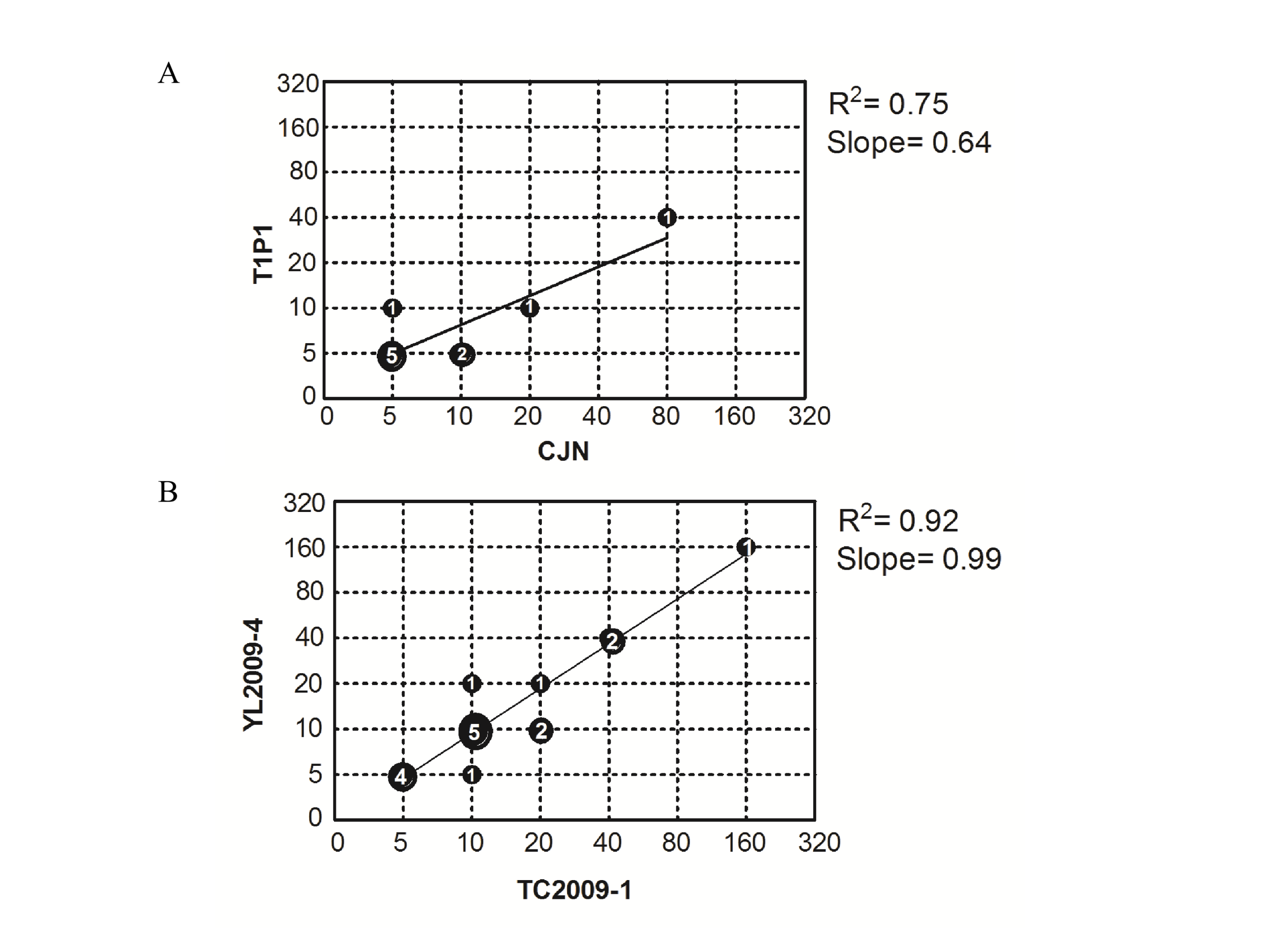

Supplement: Figure S1 — Selection of JEVs for neutralizing antibody assay. Four JEVs, GIII T1P1 (cluster II), GIII CJN (cluster I), GI TC2009-1 (cluster I), and GI YL2009-4 (cluster II), were evaluated using a panel of serum samples (N = 10 and 15 for panel A and B, respectively) by a plaque-reduction neutralization assay. The correlations of PRNT50 were determined between (A) T1P1 and CJN or (B) TC2009-1 and YL2009-4. Number in black circle corresponding to the specimen number. (TIF) [file pntd.0001834.s001.tif]

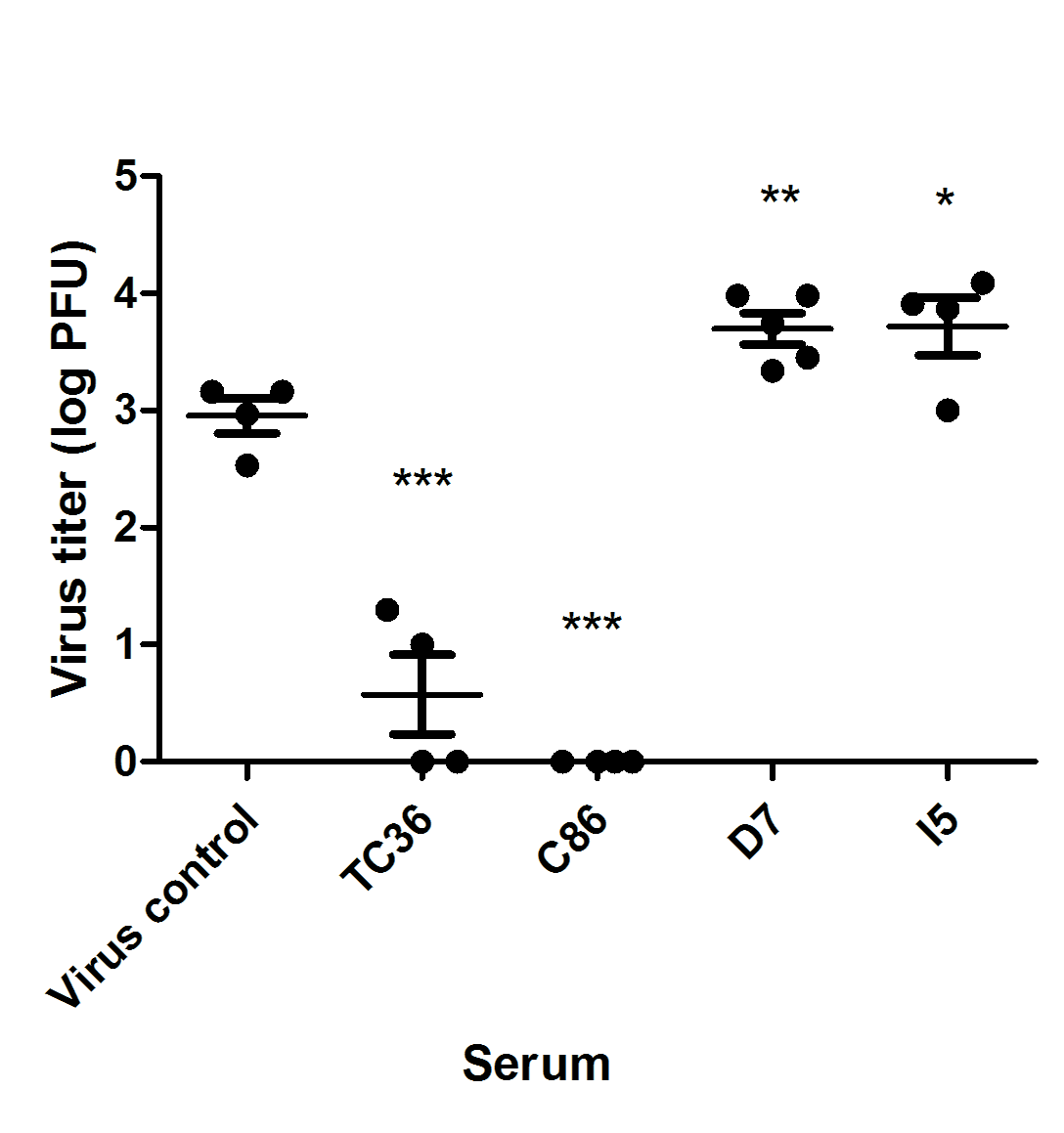

Supplement: Figure S2 — Semi-ex vivo ADE experiment. The undiluted serum was pre-incubated with 1000 PFU of TC2009-1 virus and inoculated intraperitoneally into four suckling mice per serum. Brains of inoculated sucking mice were harvested and virus titers determined by plaque forming assay in BHK-21 cells. (TIF) [file pntd.0001834.s002.tif]
