# Supplementary material for: Partially Neutralizing Potency against Emerging Genotype I Virus among Children Received Formalin-Inactivated Japanese Encephalitis Virus Vaccine
Source: PLoS Negl Trop Dis. 2012 Sep 27;6(9):e1834. doi: 10.1371/journal.pntd.0001834 (PMC3459827; doi:10.1371/journal.pntd.0001834)
Supplement: Table S1 — Amino acid variations among JEV E protein between genotype and cluster. (DOC) [file pntd.0001834.s003.doc]

Table S1. Amino acid variations among JEV E protein between genotype and cluster.

| Virus | Genotype/cluster | Amino acid position of E protein | | | | | | | | | | | | | | | | | | | | | |
| --- | --- | --- | --- | --- | --- | --- | --- | --- | --- | --- | --- | --- | --- | --- | --- | --- | --- | --- | --- | --- | --- | --- | --- |
|  |  | 36 | 51 | 65 | 83 | 123 | 129 | 138 | 176 | 186 | 222 | 227 | 242 | 276 | 290 | 306 | 327 | 340 | 348 | 366 | 418 | 486 | 499 |
| Nakayama | III/III | N | V | V | K | S | T | E | T | V | A | P | S | N | R | E | S | V | M | A | V | A | H |
| T1P1 | III/II | N | S | V | E | S | T | E | I | V | A | S | F | S | K | E | S | V | M | A | A | A | H |
| CH1392 | III/II | N | S | V | E | S | T | E | I | V | A | S | F | S | K | E | S | V | M | A | A | A | H |
| CJN | III/I | N | S | V | E | S | T | K | I | V | A | S | F | S | K | E | S | V | M | A | A | A | H |
| CC27S6 | III/I | S | S | V | E | S | T | E | I | V | A | S | F | S | K | G | S | V | L | A | A | A | H |
| CC27S8 | III/I | S | S | V | E | S | T | E | I | V | A | S | F | S | K | G | S | V | L | A | A | A | H |
| TC2009-1 | I/I | N | S | V | E | N | M | E | I | V | S | S | F | S | K | E | T | V | M | S | A | A | H |
| TC2009-2 | I/I | N | S | I | E | N | M | E | I | V | S | S | F | S | K | E | T | V | M | S | A | A | H |
| TC2009-5 | I/I | N | S | I | E | N | M | E | I | V | S | S | F | S | K | E | T | V | M | S | A | A | H |
| TC2010-1 | I/I | N | S | I | E | N | M | E | I | V | S | S | F | S | K | E | T | V | M | S | A | A | H |
| YL2010-3 | I/I | N | S | V | E | N | M | E | I | V | S | S | F | S | K | E | T | A | M | S | A | A | H |
| HL2010-1 | I/I | N | S | V | E | N | M | E | I | V | S | S | F | S | K | E | T | A | M | S | A | A | H |
| CH2010-1 | I/I | N | S | V | E | N | M | E | I | V | S | S | F | S | K | E | T | A | M | S | A | A | H |
| TN2010-1 | I/I | N | S | V | E | N | M | E | I | V | S | S | F | S | K | E | T | V | M | S | A | A | Y |
| TC2009-3 | I/II | N | S | V | E | S | M | E | I | V | S | S | F | S | K | E | T | V | M | S | A | A | H |
| YL2009-4 | I/II | N | S | V | E | S | M | E | I | V | S | S | F | S | K | E | T | V | M | S | A | A | H |
| YL2010-1 | I/II | N | S | V | E | S | M | E | I | V | S | S | F | S | K | E | T | V | M | S | A | A | H |
| CY2010-1 | I/II | N | S | V | E | S | M | E | I | I | S | S | F | S | K | E | T | V | M | S | A | A | H |
| CY2010-4 | I/II | N | S | V | E | S | M | E | I | V | S | S | F | S | K | E | T | V | M | S | A | T | H |
